# Quantum-assisted trustworthiness for the Quantum Internet

**DOI:** 10.1371/journal.pone.0319302

**Published:** 2025-04-10

**Authors:** Agustín Zaballos, Adrià Mallorquí, Joan Navarro

**Affiliations:** Engineering Department, La Salle Campus Barcelona, Universitat Ramon Llull, Barcelona, Spain; Model Institute of Engineering and Technology, INDIA

## Abstract

Device redundancy is one of the most well-known mechanisms in distributed systems to increase the overall system fault tolerance and, consequently, trustworthiness. Existing algorithms in this regard aim to exchange a significant number of messages among nodes to identify and agree which communication links or nodes are faulty. This approach greatly degrades the performance of those wireless communication networks exposed to limited available bandwidth and/or energy consumption due to messages flooding. Lately, quantum-assisted mechanisms have been envisaged as an appealing alternative to improve the performance in this kind of communication networks and have been shown to obtain levels of performance close to the ones achieved in ideal conditions. The purpose of this paper is to further explore this approach by using super-additivity and superposed quantum trajectories in quantum Internet to obtain a higher system trustworthiness. More specifically, the wireless communication network that supports the permafrost telemetry service for the Antarctica together with five operational modes (three of them using classical techniques and two of them using quantum-assisted mechanisms) have been simulated. Obtained results show that the new quantum-assisted mechanisms can increase the system performance by up to a 28%.

## Introduction

Despite extensive efforts to expand mobile network coverage worldwide through wireless communication technologies (such as 3G, 4G, LTE, and 5G), there are still regions and specific use cases that remain unable to harness the benefits of these technologies due to their intrinsic characteristics. For example, when providing reliable connectivity in remote areas poses challenges as conventional networks often fail to reach these locations, thereby necessitating more expensive alternatives like satellite communication for users in such regions. Similarly, Internet of Things (IoT) devices exposed to diverse and sometimes harsh environmental conditions and necessitated of low-power and long-range communication are typically forced to rely on well-known technologies like Narrowband IoT (NB-IoT) or Long-Range Wide Area (LoRa).

To address any potential limitations of these communication technologies and explore alternative solutions, the SHETLAND_NET research project [1] seeks to establish an experimental wireless communication network in Antarctica to support various in-field experiments. This network comprises two main layers: an access layer utilizing LoRa to connect different sensing devices, and a backhaul employing Near Vertical Incidence Skywave (NVIS) technology to provide coverage for various IoT domains within the South Shetland Islands archipelago [2]. Currently, the scientific experiments conducted on top of this communication network encounter challenges such as critical data losses, experiment delays, and costly troubleshooting efforts. These issues primarily stem from two factors: 1) the inherent unreliability of NVIS, which is highly contingent on ionosphere conditions and solar activity, and 2) the inability of the deployed devices to endure the extreme environmental conditions of Antarctica resiliently. Consequently, the overall system becomes unreliable and untrustworthy, often rendering the collected data unusable for scientific purposes.

Recently, the usage of quantum mechanics to improve the performance of this communication network has been investigated [3]. More specifically, conducted simulations have shown that quantum-assisted mechanisms could improve the overall system trustworthiness by taking advantage of devices redundancy and obtaining a performance—measured in terms of Successful Transaction Rate (STR)— that closely approaches the performance achieved by ideal centralized reputation techniques facilitated by a social IoT trustworthiness layer [3,4].

This paper proposes a new quantum-assisted mechanism based on super-additivity and superposed trajectories for quantum Internet to further increase the system’s trustworthiness and, thus, the STR. To contextualize the simulations, an IoT permafrost telemetry service deployed on top of the aforementioned Antarctic wireless communications network has been considered. According to our experience in the SHETLAND-NET research project, the target STR for a successful operation of the permafrost telemetry must be at least 60% [5]. In the proposed simulation scenario of this work, the STR is also used to evaluate the goodness of the proposed quantum-assisted mechanisms and to anticipate and identify possible weaknesses in an IoT telemetry system. This research offers, by means of simulations, the opportunity to explore potential advantages of the proposed quantum-assisted mechanisms regarding social IoT trustworthiness layer and to evaluate the benefits of altering measuring spots redundancy over the quantum Internet [6].

Overall, this paper presents three major novelties in relation to the existing scientific literature: 1) It proposes the utilization of two quantum physics theories (super-additivity and superposed quantum trajectories) that, to the best of authors’ knowledge, have not been directly applied in an engineering context to the Quantum Internet in order to enhance the trustworthiness in harsh environments; 2) the already presented simulation model in [3] has been substantially evolved by incorporating the formulation of these two quantum properties, considering that, as quantum physics theories yet to be implemented, they must adhere to the premises outlined in [7] to model the simulation environment consistently with the underlying mathematical framework discussed in [8] and [9] and, 3) a new performance analysis with a confidence interval of 99% and improving the trustworthiness results obtained in [3] by a 28% thanks to the addition of multiple scenarios developed within a new simulation setup has been conducted.

### Trustworthiness through redundancy

Trustworthiness in a communication network refers to the degree of reliability of the network’s nodes and processes in exchanging information. Redundancy, in the context of trustworthiness, is related to the presence of multiple redundant measuring spots or communication paths within the data network. Redundancies serve as a backup that ensures that if one measuring spot or path becomes compromised, other reliable options are available to maintain uninterrupted communication.

The trustworthiness of the Internet is essential for proper functioning under harsh conditions. The literature defines Internet trustworthiness through four dimensions [3,4,10,11]. Hence, the baseline trustworthiness model proposed to evaluate the Antarctic quantum Internet’s performance is layer-based and comprises four layers: data trustworthiness, network trustworthiness, social trustworthiness, and consensus layers. The data trustworthiness layer ensures the accuracy of the data provided by the source, the network trustworthiness layer ensures that packets reach their destination unaltered and on time, the social trustworthiness layer leverages objects’ social relationships to improve trust, and the consensus layer ensures that all participants agree to adopt the same versions of each data object disregard of node failures or communication delays [12]. The permafrost-related experiment in which this research is contextualized is focused on the goodness assessment of measuring spots redundancy. Measuring spots redundancy is necessary for the last two layers if we want to maximize their usefulness.

The social trustworthiness layer essentially focuses on establishing trust among network nodes through reputation-based mechanisms. These mechanisms evaluate the trustworthiness of nodes based on factors such as previous transaction feedback, indirect opinions from other nodes, transaction relevance, node centrality, computational capacity, and the nature of relationships between nodes [11]. It aims to determine which nodes are trustworthy for information exchange, maximizing the number of successful transactions. On the other hand, the consensus layer aims to achieve a decentralized general agreement among all participating nodes. It employs a voting-based mechanism that tolerates a certain percentage of byzantine nodes. Understanding the dependencies between these two layers is essential for evaluating and quantifying the trustworthiness of the communication network.

In a distributed system, consensus is achieved when all participants agree on the same (i.e., correct) value/version of a datum. Traditionally, mechanisms to enforce consensus can be grouped into two main categories: Proof-based protocols and Byzantine protocols. Proof-based protocols, typically used in blockchain applications (e.g., proof-of-stake, proof-of-work), require a considerable amount of computing power and, thus, are not suitable for IoT environments such as the ones proposed in this paper. On the contrary, Byzantine protocols just poll all the nodes of the system to reach an agreement by means of voting. This approach exchanges the required computing power of proof-based protocols by a (considerable large) number of messages to be exchanged through the communications network. Therefore, Byzantine protocols are a more suitable alternative for this work as the communication network is generally available in IoT environments. A further comparison and analysis of well-known Byzantine protocols, including Practical Byzantine Fault Tolerance and RAFT (Reliable, Replicated, Redundant, And Fault-Tolerant) can be found in [13,14]. Despite the widespread use of these consensus protocols, existing trustworthiness approaches in the literature often focus on specific aspects, neglecting the interdependencies between different trustworthiness categories. This oversight can lead to misinterpretations of a service’s trustworthiness issues and result in ineffective solutions. To address this gap, we designed a new model capable of evaluating the trustworthiness of IoT systems across all four major trustworthiness domains. This model aims to better predict and identify potential weaknesses, helping to enhance the reliability of IoT telemetry systems [11].

The proposed quantum trustworthiness model combines reputational and consensus quantum algorithms for optimal trustworthiness performance. The previous work [3] explores the use of the Fast Quantum Consensus algorithm to achieve instantaneous coordination of communication parties, avoiding traffic congestion’s negative impact. Indeed, in traffic-overwhelmed scenarios, voting-based consensus algorithms can lead to increase traffic congestion, decreasing the Internet’s trustworthiness. As a result, [3] shows that the implementation of a management plane driven by quantum consensus that takes advantage of the inherent redundancy of measurement spots, brings a 16% improvement in terms of trustworthiness compared to its classical (non-quantum) alternative. This previous work also shows that the proposed distributed fast quantum consensus protocol barely improves by 5% the reference value obtained through centralized reputation algorithms (social trustworthiness layer over classical Internet).

### Quantum-assisted trustworthiness

Given the observed improvement in trustworthiness achieved by quantum consensus compared to classical consensus (specifically, Fast Quantum Consensus over the Practical Byzantine Fault Tolerance protocol), and with the aim of getting closer to the maximum reference value provided by the classical social trustworthiness layer [4], this paper poses the following research question: What enhancements could be achieved by applying quantum-assisted mechanisms, such as super-additivity and superposed trajectories, to the social trustworthiness layer, which sets an upper bound on STR in the classical Internet?

In this way, our objective is to explore the potential benefits derived from incorporating quantum features into the social trustworthiness layer. To this end, we have evaluated how leveraging quantum properties, such as super-additivity and superposed trajectories, can further improve the performance and reliability of communication protocols beyond what is currently attainable in classical systems. By doing so, we aim to provide valuable insights into the feasibility and potential advantages of integrating quantum enhancements, thereby paving the way for advancements in Antarctica telemetry and potentially other domains where reliability and efficiency are critical.

Getting into the matter, quantum super-additivity refers to the phenomenon where the total amount of information that can be transmitted through a quantum channel is greater than the sum of the individual capacities of its subchannels. In other words, by combining two or more quantum channels, one can achieve a greater amount of information transmission than by using the channels separately [8]. This concept is related to the properties of entanglement in quantum mechanics. Entanglement is a type of correlation between quantum systems that can exist even when they are physically separated. This means that manipulating the entangled systems in a certain way makes it possible to transmit more information than can be achieved by transmitting the systems separately. In fact, when two entangled qubits are sent through separate channel events, the amount of information that can be transmitted through each channel utilization individually is limited by the channel’s capacity. However, by combining both channel events, the entanglement between the qubits can increase the total amount of information that can be transmitted. The phenomenon of quantum super-additivity has been demonstrated experimentally and has important implications for quantum communication and information theory [8].

On the other hand, quantum trajectories refer to a theoretical framework for describing the time evolution of qubits in terms of individual trajectories in the space of quantum states. In traditional quantum mechanics, the evolution of a qubit is defined by a wave function, which gives the probability amplitude for the system to be in a particular state. Quantum trajectory theory extends the wave function description by introducing the concept of quantum state reductions, which occur when a qubit interacts with its environment. In [9], authors demonstrate experimentally that superposing multiple quantum trajectories can enhance the performance of quantum communication protocols. They show that combining multiple trajectories in a qubit can increase the amount of information that can be transmitted through a quantum channel. The key is that by superposing multiple quantum trajectories, it is possible to exploit the inherent quantum mechanical properties of entanglement and nonlocality to enhance the performance of quantum communication protocols.

Both quantum mechanisms are a consequence of the non-classical correlations that can exist between different quantum channels’ events. The success probability of a quantum channel’s event could be enhanced by the existence of another entangled quantum channel’s event. This means, for example, that by adding a weak channel to a strong channel, the capacity of the strong channel can be increased. In fact, this can be used in quantum channels in serial by transmitting a quantum state one after another. This can increase the capacity of the quantum channel and reduce the impact of noise or errors introduced by the environment. It highlights the non-classical properties of quantum channels and the potential for quantum protocols to achieve higher trustworthiness than classical protocols.

To implement super-additivity and superposition of trajectories in serial or parallel quantum communications, it is necessary to design protocols that can exploit the non-classical properties of quantum channels, such as entanglement and non-additivity [15]. These protocols can be complex and may require advanced quantum technology, but they have the potential to improve the performance of quantum communication in serial significantly. Even though the enabling core technologies to physically implement a quantum Internet are still under development, this should not be a blocking barrier to research into the possibilities that quantum Internet could offer. In fact, it is worth mentioning that while the implementation of the low layers (i.e., Layer 1 – physical and Layer 2 – Data Link) of quantum networks nowadays require expensive (and very experimental) hardware, upper-layer protocols (i.e., Layer 3 – Network, and beyond) such as the ones presented in this work can be tested via simulations when the simulation environment is consistently modeled with the practical physical facts [7].

This suggests that by leveraging these unique properties of quantum systems, it should be possible to develop a new and more efficient social trustworthiness layer for secure communication.

### Simulation environment

**Fig 1:**
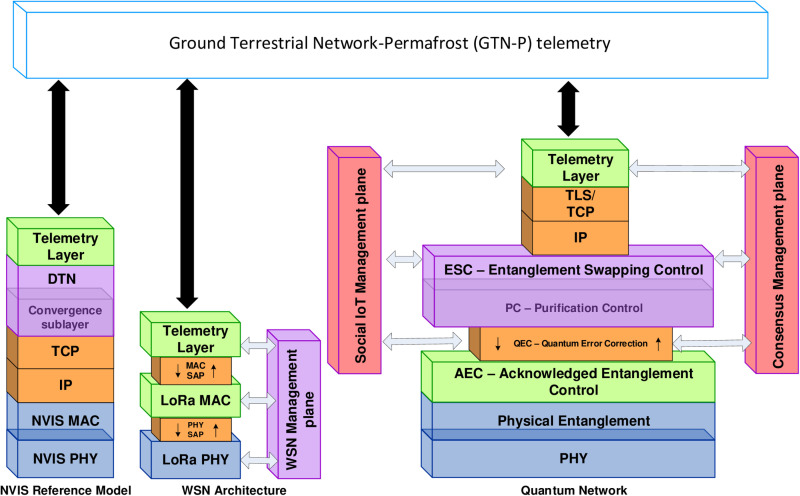
Protocol architecture proposed for our experimentation.

In order to obtain comparable results with the previous work presented in [3], the same simulation setup has been selected. That is, the scenario of permafrost telemetry in Antarctica has been modeled and implemented in the Riverbed Modeler simulator [6]. To conduct simulation tests and assess the outcomes using our proposed quantum-assisted trustworthiness model, we have modeled communication elements, including the classical communication media, such as the physical and link layers of LoRa and NVIS technologies. Moreover, we have incorporated the link layer mechanisms of the quantum Internet, including quantum pre-processing, quantum post-processing, and entanglement generation and distribution. The modeled scenario also includes the telemetry application layer, the detection of faulty behavior of byzantine nodes, and the management of social trust. All of them integrated with the quantum management plane and the quantum trustworthiness management plane (see Fig 1). It is worth highlighting that a Delay Tolerant Network (DTN) is needed to be placed below the telemetry layer to fight against the effects of the NVIS channel dynamic availability, which is known to be motivated by the unstable behavior of solar activity and the ionosphere itself [16]. This DTN layer is devoted to ensures that the communications network can operate efficiently within the range of 70% to 100% NVIS availability. Further details regarding the implementation of the simulation models and their associated finite state diagrams that model the permafrost telemetry service use case that has been also used in this work can be found in [11]. The main traits of the simulation environment are summarized in what follows:

Our model for the telemetry service uses long-distance quantum communication by using entanglement-based physical technologies, which is used exclusively in the reputation and consensus phase. The experiments carried in this work to analyze improvements in global trustworthiness follow the guidelines that Dahlberg et al. [17] stipulate for an MD scheme (Measure Directly use case) in which there is no quantum memory to store entanglement, and qubits are immediately generated and transmitted. This means that the errors brought by the quantum noise, which in the simulated experiment are modeled following the recommendations of [3,18], are subsumed in the global error figure of the system. Therefore, the decoherence effect is used to refer to any process that affects the qubits, including perturbations, noise, and imperfections. Thus, the error model used in our proposal adds the decoherence effects as quantum multiplicative imperfections and is measured with the help of decoherence times which depend on the technology used for qubits [7,19]. Quantum channel capacity can be easily appraised by means of the relative entropy of entanglement by combining point-to-point quantum communications with classical network algorithms. In this way, [20] extends routing problems from classical to quantum communications. Anyway, in the MD scheme used in this work, quantum operations are completed before the qubits lose information. If $\left\{\gamma_{k}\right\}$ are the decay rates for ($\hat{\text{x}}$,$\hat{\text{y}}$,$\hat{\text{z}}$)-coordinates in the qubit representation, the mixed state model time evolution can be modeled as the following density matrix (1):


$$\text{ρ}\left(\text{t}\right)=\left(\begin{array}{cc} \text{ρ}{\left(\text{t}\right)}_{00} & \text{ρ}{\left(\text{t}\right)}_{01}\\ \text{ρ}{\left(\text{t}\right)}_{10} & \text{ρ}{\left(\text{t}\right)}_{11} \end{array}\right)=\frac{1}{2}\cdot \left(\begin{array}{cc} 1+z\left(0\right)\cdot e^{-2t\left(\gamma_{x}+\gamma_{y}\right)} & x\left(0\right)\cdot e^{-2t\left(\gamma_{y}+\gamma_{z}\right)}-jy\left(0\right)\cdot e^{-2t\left(\gamma_{x}+\gamma_{z}\right)}\\ x\left(0\right)\cdot e^{-2t\left(\gamma_{y}+\gamma_{z}\right)}+jy\left(0\right)\cdot e^{-2t\left(\gamma_{x}+\gamma_{z}\right)} & 1-z\left(0\right)\cdot e^{-2t\left(\gamma_{x}+\gamma_{y}\right)} \end{array}\right)$$ (1)

To model the social trustworthiness layer, a reputation mechanism [21] is adopted to identify those reliable nodes—that are physically redundant—that can actively participate in the telemetry service. The performance metric for this layer’s contribution to the overall system trustworthiness is the Successful Transactions Rate (STR). This layer employs concentrator elements to calculate nodes’ reputation according to the feedback obtained from prior transactions, establishing both short-term and long-term reputation assessments [10]. It is worth noting that all transactions are treated with the same weight (i.e., a heartbeat frame and a data frame with the measured data have the same impact on calculating the reputation), and all nodes/sensors are initialized uniformly in the simulation environment. Employing a virtual centralized method (i.e., does not consume communication bandwidth) in the simulation allows us to determine the highest possible trustworthiness in a traditional (i.e., without quantum Internet) network.

A reputation mechanism has been employed to model the social trustworthiness layer, aiming to identify the reliable nodes involved in redundant telemetry. Within this layer, concentrator elements calculate reputation by considering the feedback received from previous transactions, forming both short-term and long-term opinions. In our simulations, all transactions are assumed to have equal weight, and the sensors are initialized in a similar manner. By utilizing a centralized and bandwidth-efficient implementation within the simulation scenario, we can infer the maximum achievable trustworthiness from a classical Antarctic network deployment with redundancy. It is important to note that the performance indicator used by this layer to assess its contribution to trustworthiness is also the Successful Transactions Rate (STR).

A consensus trustworthiness layer has been included to enable all nodes to reach a unified state and adopt the same data version for each measurement, which aims to drive the system to a general agreement situation. The implemented consensus protocol, widely used in IoT environments [4,11], uses a voting-based mechanism to tolerate byzantine nodes. Although this layer is committed to improve the system trustworthiness in a deployment with physically redundant measuring spots, the bandwidth consumption associated to its inherent voting process must be considered. In situations of excessive traffic jams, particularly with a high number of redundant sensors, the system may experience congestion. In this regard, to fight this potential issue a Practical Byzantine Fault Tolerance mechanism [22] has been implemented in the simulation environment.

A Quantum Consensus Management plane (see Fig 1), which is responsible to reach a decentralized consensus by means of a quantum protocol—thus reducing message exchanges in the voting-based process. This is realized through the Fast Quantum Consensus algorithm, as detailed in the experiment described in [23,24]. Similarly, a Quantum Social Management plane (see Fig 1) is in charge of the sensor reputation management by using the super-additivity property and superposing multiple quantum trajectories as referenced experiments [8,9].

Finally, the physical and link layers have been modeled to be aligned with quantum mechanics [15] by enabling the generation and distribution of entangled pairs. The routing protocol incorporates a logical point-to-point quantum topology (i.e., path optimization based on quantum metrics is unnecessary). Considering its similarities to circuit-switched schemes, the transport layer incorporates a proactive congestion control mechanism for the quantum Internet. In short, the parameters employed for the classical Internet, specifically corresponding to LoRa and NVIS deployment, are promoted from the ones outlined in [4,11]. In the simulated scenarios, the main goal is to assess the impact of quantum-assisted mechanisms to approach the optimal global figure of merit (STR). Thus far, the classical social layer has proven to be the paradigm yielding the highest trustworthiness in the Antarctic telemetry use case.

The trustworthiness model [11,25] used in this research integrates 4 heterogeneous dimensions (also referred to as layers): Social, Data, Consensus, and Network. Each dimension uses its own metric and improvement mechanism to improve the overall trustworthiness (see Table 1). Specifically, the Social Trustworthiness Layer considers the trust between neighboring nodes by measuring the number of successful data exchanges among nodes over time. Similarly, the Data Trustworthiness Layer considers the trust of data objects by measuring the proportion of received correct data over the overall received data. Also, the Consensus Layer considers the trust of all nodes within the scenario by detecting faulty or malicious nodes. Finally, the Network Trustworthiness Layer considers the trust of the communication network by measuring its reliability (i.e., counting the number of packets that have been successfully delivered over time).

**Table 1:** Background information about the trustworthiness model and formulation.

Layer	Metric	Formula	Countermeasure mechanisms (e.g.,)	Countermeasure Quantum mechanisms
**Social Trustworthiness Layer**	Successful Transaction Rate (STR) Number of successful transactions (ST) Total transactions (TT)	$STR=\frac{ST}{TT}$	Implement reputational systems and ostracize low-reputation nodes to improve transaction success	Social IoT Management Plane (Fig 1) uses superadditivity and multiple quantum trajectories [8,9] in the feedback transmission from nodes to calculate sensor reputation when there is redundancy.
**Data Trustworthiness Layer**	Faulty Sensing Ratio (FSR) Number of faulty sensed values (FSV) Total sensed values (TSV)	$FSR=\frac{FSV}{TSV}$	Use of corrective methods (e.g., thresholds, historical comparisons) to detect and fix faulty sensed data	Quantum Error Correction [19] Quantum Error Correction Codes [18] <Not implemented in WSN, see Fig 1>
**Consensus Layer**	Byzantine Node Tolerance (BNT) Number of byzantine nodes the system can tolerate (Nb) Total number of nodes participating in consensus (Nt)	$BNT=\frac{Nb}{Nt}$	Use Byzantine Fault Tolerance (PBFT, RAFT) or voting-based consensus protocols to improve agreement across nodes	Fast Quantum Consensus approach [24] to reduce the overwhelmed effect of the consensus mechanism. Consensus Management Plane (Fig 1) takes profit of multiple quantum trajectories [9] due to channels combined in a quantum trajectory can transmit more information [8].
**Network Trustworthiness Layer**	Packet Delivery Ratio (PDR) Number of packets received (Pr) Number of packets sent (Ps)	$PDR=\frac{Pr}{Ps}$	Use transmission coding (e.g., FEC), routing protocols, DTN architectures, and QoS mechanisms to ensure delivery	Superadditivity and multiple quantum trajectories [8,9] <not implemented for sensed data flow>

The simulation scenario consists of five NVIS concentrator nodes, each including its own LoRa coverage area and equipped with sensors for telemetry. To evaluate the effectiveness of the quantum social and quantum consensus layers, redundancy in the measuring spots has been simulated at each GTN-P node to capture multiple measurements of the same data. Furthermore, each concentrator node has a variable number (ranging from 0 to 5) redundant sensor nodes, allowing us to assess the system robustness (i.e., tolerance) when nodes are exposed to byzantine behaviors. To evaluate the performance evolution—in terms of scalability—of the aforementioned quantum-assisted protocols, the simulated permafrost telemetry service in the Antarctica has been simulated with 32 and 64 permafrost measuring spots. Each test has been repeated 30 times, resulting in a total of up to 115,000 tests in which the average value of the STR has been computed. The obtained results that are exhibited have a confidence interval of 99%.

## Results and Conclusions

After the completion of the simulations, the STR was calculated by averaging the obtained values. As the simulations aimed to cover the timespan between two successive Antarctic campaigns, a simulation period of 400 days was selected. In addition, the simulated scenarios include various values for the byzantine fault probability (Pb_0_), ranging from 10^-1^ to 10^-3^. The selection of values within this interval is aimed to model the usage of different on-field devices (e.g., sensor sources, batteries) and see their impact of the system’s trustworthiness. In this way, low values of Pb_0_ are assigned to high quality and reliable devices while high values of Pb_0_ are assigned to poor quality or unreliable devices.

**Fig 2:**
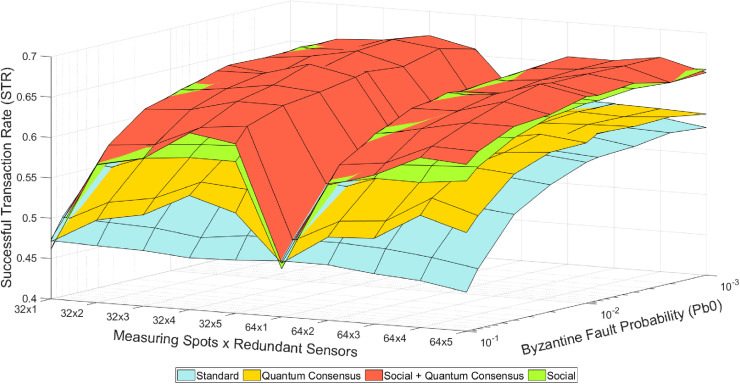
Colored mesh for STR analysis (classical Internet and quantum-assisted consensus) obtained in [3].

**Fig 3:**
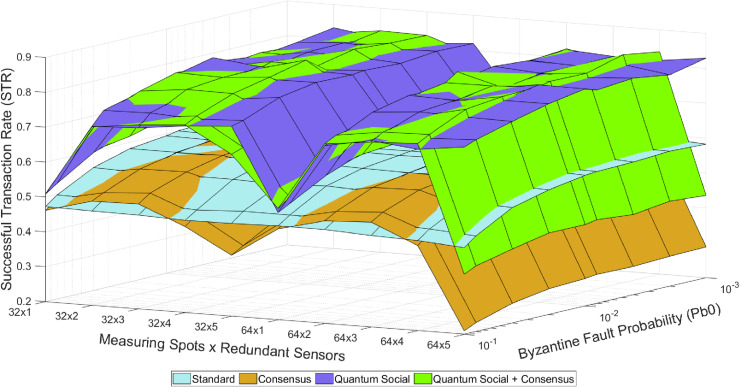
Colored mesh for STR analysis (classical Internet and quantum-assisted social trustworthiness).

**Fig 4:**
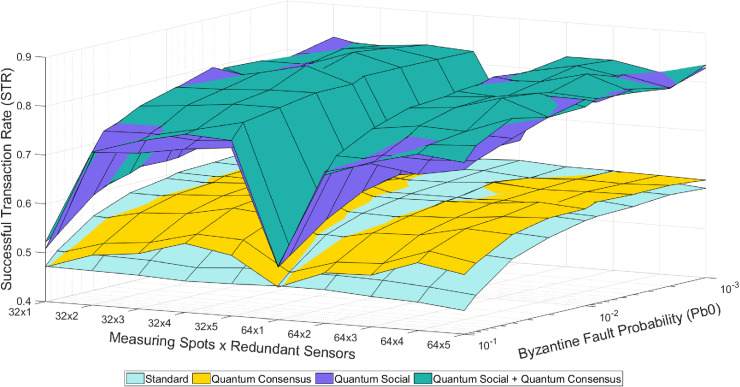
Colored mesh for STR analysis (classical baseline and quantum-assisted mechanisms combination).

There are five different operational modes based on the utilization of redundant-related mechanisms (see Figs 2–4). These five modes, as well as their combination, represent different strategies for handling redundancy and ensuring trustworthiness in the system:

1) In the “Standard” mode, nodes transmit data directly to the control center immediately after gathering it, without any further interaction. As in this mode none of the consensus protocols are used, the effects and benefits associated to the physical redundancy of measuring spots have very little impact on the STR.
2) In the “Social” mode, are enabled to execute the social trustworthiness layer implemented in the Social IoT Management plane (see Fig 1). Thanks to the addition of this module, (untrustworthy) nodes providing wrong or inconsistent data are detected and isolated (i.e., ignored) from the data broadcasting rounds.
3) In the “Consensus” mode, nodes are programmed to reach an agreement on the correct sensed value before sending it to the control center. That is, they interact between each other to compare their sensed value with the ones sensed by their neighbors. Thanks to the implementation of the aforementioned Practical Byzantine Fault Tolerance protocol [4,11] (see Section IV), the system is able to tolerate byzantine behaviors in nodes.
4) In the “Quantum Consensus” mode, nodes use the aforementioned Fast Quantum Consensus protocol [23,24] (see Section IV) to reach an agreement on the correct measured data. Note that this protocol takes advantage of the properties of quantum mechanics to drastically reduce the number of messages required to synchronize data in nodes.
5) Finally, in the “Quantum Social” mode, nodes develop the social trustworthiness layer by incorporating super-additivity and superposed multiple quantum trajectories to get closer to the theoretical optimum value of the used figure of merit, as explained before [8,9].

Figures 2–4 illustrate the overall pattern of STR values observed in our simulation results. In each figure, a grid with an X ×  Y dimension is created, representing all possible combinations of simulation parameters. Here, X represents the number of different Pb_0_ values (i.e., byzantine fault probability), and Y represents the number of different sensors (i.e., measuring spots and redundancy). For each point on this grid and each mode combination, the average value of the trustworthiness STR metric is calculated. By connecting the STR values for adjacent points on the grid, a mesh is formed that encompasses all the STR values. This trustworthiness mesh provides a comprehensive representation of the trustworthiness across different simulated scenarios.

In general, as the “Byzantine Fault Probability” increases along its axis, the STR decreases because a higher Pb_0_ leads to more faulty sensed values. On the other hand, as the “Redundant Sensors × Sensor Clusters” increases along its axis, the STR also decreases because of the introduction of more devices to the network, resulting in more packet losses due to network congestion. These results illustrate the benefits of using quantum-assisted mechanisms still within the constraints imposed by the selected use case (i.e., the number of redundant sensors per cluster is limited to 5, so only one byzantine node is theoretically tolerated per consensus group and the simulation scenario includes 32 and 64 permafrost measuring spots). In this way, the “Redundant Sensors × Sensor Clusters” axis has 10 discrete points labeled as [32 ×  N, 64 ×  N], where N ranges from 1 to 5. The observed behavior remains consistent, as the STR values recover when transitioning from the “32 × 5” to the “64 × 1” point due to fewer nodes being introduced, resulting in fewer packet losses caused by network congestion. Figures 2–4 depict the trustworthiness mesh for the analyzed modes. After describing the method for interpreting these figures, one can easily understand the qualitative results of the tests.

Certainly, the insights derived from these results can be extrapolated to other scenarios with similar characteristics. For instance, it is straightforward to imagine IoT domains populated by different (redundant) devices that exchange critical data through an unreliable communications network [26–28]. Although the experiments conducted so far (see Figures 2–4) have limited the maximum number of redundant measuring spots to 5, it is still interesting to assess the behavior of the STR in a more general scenario where this number goes beyond. However, it is worth considering that the potential STR gain due to an increased redundancy will come at the price of buying more sensors.

**Fig 5:**
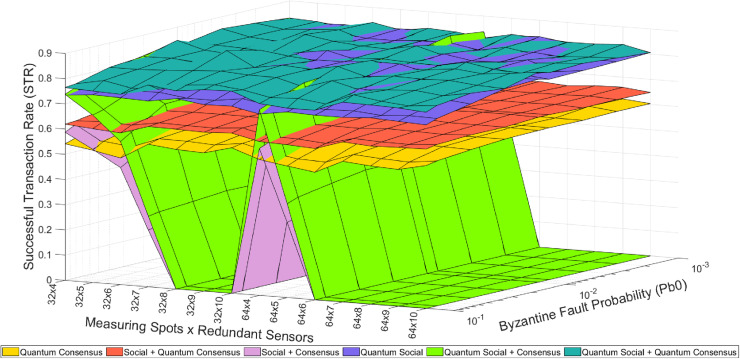
Trustworthiness mesh varying the number of redundant sensors from 4 to 10.

In this regard, a generalized version of the system has been simulated and the obtained results are depicted in Fig 5. As it can be seen, this new simulation aims to assess the STR when the number of redundant measuring sensors ranges from 4 to 10. It can be seen that this figure compares the results between the quantum-assisted simulated modes and the classical ones (i.e., we can observe the impact of quantum-assisted modes in contrast to classical modes).

In this new experiment, it becomes evident that utilizing 5 or more redundant sensors with classical consensus leads to a considerable degradation in the achieved STR, dropping below 0.6. Furthermore, the STR plunges to 0 in two configurations: either (a) when the number of measuring spots is 32 and the number of redundant sensors is higher than 7 and or (b) when the number of measuring spots is 64 and the number of redundant sensors is higher than 6. This means that under these configurations the system is unable to successfully send messages between nodes due to the network congestion brought by (1) the poor capacity of the network (i.e., low bandwidth) on transmitting messages (2) the high number of message senders and receivers.

In contrast, the proposed quantum consensus protocol greatly mitigates this effect by drastically reducing the number of data synchronization messages among nodes [3]. Consequently, the network remains less congested even in a situation with an increased number of nodes and, thus, allowing the STR to stay above the 0.6 threshold. This quantum approach also slightly improves the overall trustworthiness compared to the reference mechanism based on the centralized social trustworthiness layer. Even more impressive is the result obtained using the quantum social mode. While we were previously concerned about losing an acceptable STR level for the permafrost use case (i.e., 60%) due to channel capacity, this new mode exceeds expectations by far. Although the quantum consensus reaches STR levels close to the reference value (i.e., the achieved with the social trustworthiness mode), the quantum social mode goes beyond the maximum reference value by over 27%, thus raising the reference value from a 60% STR to 85% in the simulated scenario.

Examining the average STR results from these simulations (Table 2) a significant improvement when using quantum-assisted protocols can be perceived. For instance, it can be seen that in the initial battery of experiments (see Figures 2–4), the “Quantum Consensus,” “Quantum Social,” and “Quantum Social + Quantum Consensus” methods demonstrated improvements over their classical counterparts by 1.3%, 26%, and 39%, respectively. Conversely, in the second round of tests (see Fig 5) with a higher number of redundant sensors, the “Quantum Consensus” mode shows a 2% improvement over the “Consensus” mode, the “Quantum Social” mode enhances the “Social” mode by 27%, and the “Quantum Social + Quantum Consensus” mode outperforms the “Social + Consensus” mode by 28%. These results underscore the fact that quantum-assisted mechanisms become increasingly valuable as the number of redundant nodes grows. Therefore, quantum-assisted mechanisms like the ones explored in this paper have the potential to facilitate the use of larger-scale scenarios with a greater number of sensors and measurement spots.

**Table 2:** Maximum and average STR for each operation mode in the first round (basic permafrost use case) and second round of tests (by following the generalization approach).

	Maximum (use case)	Average (use case)	Maximum (extended)	Average (extended)
Standard	0.610	0.562	0.610	0.550
Social	0.659	0.617	0.659	0.626
Consensus	0.603	0.518	0.603	0.289
Social + Consensus	0.673	0.586	0.673	0.332
Quantum Consensus	0.611	0.577	0.615	0.584
Social + Quantum Consensus	0.675	0.624	0.691	0.637
Quantum Social	0.833	0.755	0.840	0.774
Quantum Social + Consensus	0.853	0.715	0.853	0.406
Quantum Social + Quantum Consensus	0.852	0.765	0.864	0.788

Beyond the improvements introduced in [3] about the fast quantum consensus, and more specifically in the main contribution of this article regarding the quantum social trustworthiness control plane: The non-additivity property of quantum channels is a fundamental property of quantum mechanics that distinguishes it from classical physics. In classical physics, the capacity of a communication channel is determined by its physical properties, such as bandwidth and noise level. These properties are additive, which means that the combined capacity of two channels is the sum of their individual capacities. In contrast, quantum channels exhibit non-additivity, which means that the combined capacity of two quantum channels can be greater than the sum of their individual capacities. This property arises from the non-classical correlations between quantum systems, such as entanglement. The non-additivity property of quantum channels can be modeled as stated in the simulation setup chapter. It also highlights the non-classical properties of quantum mechanics and the potential for quantum-assisted technologies to achieve higher performance than classical technologies.
